# F102. CHANGE IN PATTERNS OF CANNABIS AND OTHER SUBSTANCE USE OVER TIME IN EARLY PSYCHOSIS- EXAMINING THE EFFECT OF DEVELOPMENT OF PSYCHOSIS

**DOI:** 10.1093/schbul/sby017.633

**Published:** 2018-04-01

**Authors:** Musa Sami, Michael Lynskey, Sagnik Bhattacharyya

**Affiliations:** King’s College London

## Abstract

**Background:**

Understanding how the onset of psychosis affects patterns of substance use would inform the development of effective interventions. To date no study has compared substance misuse patterns over a life-course to a comparable control group to determine how patients who develop psychosis modify substance misuse patterns.

**Methods:**

In a well-characterised clinical cohort of patients with psychotic disorders (n=257) we compared frequency of use of most common substances before and after development of psychotic disorder using a within subjects design. Using a between-subjects design we compared patients who had ever used cannabis (n=194) to a control non-clinical cohort of cannabis users (n=1055) over comparable periods in life, accounting for the effects of age, gender, other substance use and location.

**Results:**

Patients reduced frequency of consumption of cannabis, alcohol, cocaine and ecstasy (p<=0.001, all comparisons) but not tobacco or crack cocaine. Since adolescence, compared to controls, patients were more likely to reduce cannabis frequency (OR 2.3, p<0.001) and less likely to have increased cannabis frequency (OR 0.2, p<0.001). Patients with psychosis were more likely to have used heavily earlier, with a greater proportion using cannabis more than once weekly, using more potent forms of cannabis, and reporting solitary use up to age 16. However, with regards to current use, controls were currently using more heavily across these parameters than patients.

**Discussion:**

Patients who develop psychosis decrease substance consumption after adolescence compared to other non-psychotic substance users. A history of heavy early use of cannabis may be a tractable target for intervention.

